# Hydroxyzine Effects on Post-Lanosterol Biosynthesis in Smith–Lemli–Opitz Syndrome (SLOS) Models

**DOI:** 10.3390/biom15040562

**Published:** 2025-04-10

**Authors:** Zeljka Korade, Allison C. Anderson, Marta Balog, Keri A. Tallman, Ned A. Porter, Karoly Mirnics

**Affiliations:** 1 Department of Pediatrics, Biochemistry and Molecular Biology, College of Medicine, University of Nebraska Medical Center, Child Health Research Institute, Omaha, NE 68198, USA; zeljka.korade@unmc.edu; 2 Munroe-Meyer Institute for Genetics and Rehabilitation, University of Nebraska Medical Center, Child Health Research Institute, Omaha, NE 68198, USA; allison.anderson@unmc.edu (A.C.A.); mbalog@mefos.hr (M.B.); 3 Department of Medical Biology and Genetics, Faculty of Medicine, Josip Juraj Strossmayer University of Osijek, 31000 Osijek, Croatia; 4 Department of Chemistry, Vanderbilt Institute of Chemical Biology and Vanderbilt Kennedy Center for Research on Human Development, Vanderbilt University, Nashville, TN 37240, USA; keri.a.tallman@vanderbilt.edu (K.A.T.); n.porter@vanderbilt.edu (N.A.P.)

**Keywords:** 7-DHC, DHCR7, desmosterol, sterol biosynthesis, LC-MS/MS

## Abstract

Smith–Lemli–Opitz syndrome (SLOS) is a developmental disability arising from bi-allelic pathogenic variants in the 7-dehydrocholestrol reductase (DHCR7) enzyme and the accumulation of 7-dehydrocholesterol (7-DHC). 7-DHC spontaneously oxidizes and gives rise to cytotoxic oxysterols. Our recent high-throughput screening on *Dhcr7*-deficient Neuro2a cells identified hydroxyzine (HYZ) as a medication that could counteract the high levels of 7-DHC. We assessed the effects of HYZ in *Dhcr7*-deficient Neuro2a cells, neuronal cultures and glial cultures from Dhcr7^T93M/T93M^ transgenic mice, and human dermal fibroblasts from patients with SLOS. LC-MS/MS biochemical analyses revealed a strong modulatory effect of HYZ on post-lanosterol biosynthesis across all four SLOS models. However, the HYZ-induced biochemical changes were complex, dose-dependent, and variable across the four SLOS models. *Dhcr7*-deficient Neuro2a cells showed decreased 7-DHC, 8-dehydrocholesterol (8-DHC), and desmosterol (DES) levels (all *p* < 0.01), while neuronal and glial cultures from Dhcr7^T93M/T93M^ transgenic mice reported 8 significantly altered analytes (all *p* < 0.001). Human dermal fibroblast from patients with SLOS reacted to HYZ exposure with significantly decreased 7-DHC, 7-dehydrodesmosterol (7-DHD), and dihydrolanosterol (DHL) levels (*p* < 0.001), coupled with elevation in zymosterol (ZYM), zymostenol (ZYME), and 8-DHC (*p* < 0.001). Further evaluations are required to determine if the potentially beneficial effects of decreased 7-DHC, 7-DHD and DHL levels in SLOS models and patient biomaterials are counteracted by the rise in other post-lanosterol intermediates.

## 1. Introduction

Smith–Lemli–Opitz syndrome (SLOS) is a complex intellectual and developmental disability arising from two pathogenic variants in the dehydrocholesterol reductase 7 (DHCR7) gene [1,2,3,4,5,6,7,8,9,10]. The genetic inhibition of the DHCR7 gene reduces the conversion of 7-dehydrocholesterol (7-DHC) to cholesterol, resulting in the accumulation of 7-DHC in virtually all cells of the body, including the brain [1,11,12,13,14,15,16,17]. As 7-DHC is the most oxidizable lipid known to date [18,19,20,21], it gives rise to a host of 7-DHC-derived oxysterols [22,23,24], which are toxic for developing cells [25,26,27,28,29,30]. Thus, the SLOS phenotype arises by two simultaneous adverse molecular events during development: reduced cholesterol levels and increased 7-DHC levels (with a corresponding rise in 7-DHC-derived oxysterols) [20,31,32,33,34,35,36,37].

The cholesterol synthesis pathway is conserved across mammalian species, includes over 30 dedicated enzymes, and provides substrates for many cellular metabolic pathways [38,39,40]. After lanosterol synthesis, cholesterol biosynthesis proceeds via two separate, but interactive branches called the Kandutsch–Russell and Bloch branches. The utilization of these branches depends on the cell type [41]. Endogenous sterol synthesis in the developing brain is complex, where both neurons and glia preferentially utilize the Bloch biosynthetic pathway, with DES being the immediate precursor of CHOL [42]. In SLOS, the inhibition of DHCR7 affects both the Bloch and Kandutsch–Russel branches, leading to increased levels of 7-DHC, 7-DHD, and 8-DHC and decreased levels of desmosterol and cholesterol [34]. For this reason, comprehensive analysis of post-lanosterol intermediates is essential to fully evaluate the complexity of SLOS phenotype.

There is currently no effective treatment of SLOS. Knowledge-based treatments such as dietary cholesterol supplementation have been less effective than desired [43,44,45,46]. The body and brain cholesterol pools are separated by the blood–brain barrier, and the brain synthesizes its own cholesterol [38,39]; thus, the dietary supplementation of cholesterol does not have a beneficial effect on the developing brain [43,47]. As a result, the search for effective treatments continues, with a particular focus on counteracting the toxic effects of 7-DHC. One possible very appealing avenue was the screening of approved medications for clinical use for their ability to reduce 7-DHC levels. In our recent study, using the NIH Clinical Collection library, we found that of the 727 medications approved for human use, over 30 compounds decreased 7-DHC in *Dhcr7*-deficient Neuro2a cells [48]. Based on the magnitude of 7-DHC level reductions and mechanism of action, our screening identified hydroxyzine (HYZ) as one of the leading candidates for potential treatment of SLOS. HYZ is an antihistamine, with multiple indications for treatments of adults and children [49,50,51,52]. These indications encompass itching caused by allergic skin reactions, relief of anxiety and tension, and use as sedative before and after general anesthesia. It is primarily a potent H1 receptor blocker, with more than 50 years of history in FDA-approved human clinical use [53,54,55,56,57]. HYZ is generally considered a well-tolerated and safe medication, with an approximate 20 h half-life [58,59]. It is metabolized in the liver by CYP3A4 and CYP3A5 [60].

In this study, we asked if HYZ could potentially be repurposed as a knowledge-based treatment for patients with SLOS. We hypothesized it could reduce high 7-DHC levels seen in SLOS models and that it would normalize the post-lanosterol biochemical profile. We used LC-MS/MS assessments of post-lanosterol intermediate levels to test HYZ effects on the hallmark biochemical features of SLOS. The analyses were performed on four different SLOS models: *Dhcr7*-deficient Neuro2a cells [61], neuronal and glial cultures from Dhcr7^T93M/T93M^ transgenic mice (a validated SLOS transgenic mouse model) [31] (REF), and human dermal fibroblasts from patients with SLOS [62].

## 2. Materials and Methods

### 2.1. Chemicals

Unless otherwise noted, we utilized Sigma-Aldrich Co. (St. Louis, MO, USA) for all chemical supplies. HPLC-grade solvents were obtained from Thermo Fisher Scientific Inc. (Waltham, MA, USA). Hydroxyzine was procured from Selleck Chemicals (Houston, TX, USA). The sources of sterol standards used (both natural and isotopically labeled) were from Kerafast, Inc. (Boston, MA, USA).

### 2.2. Neuro2a Cell Cultures

Neuro2a cells were obtained from ATCC. *Dhcr7*-deficient Neuro2a cells, generated by stable Dhrc7-shRNA-transfection, have been extensively validated as described previously [61]. They were kept in EMEM supplemented with L-glutamine (Gibco cat # A2916801), 2 mM, 10% FBS (Seradigm cat # 97068-085) at 37 °C and 5% CO_2_. Cells were subcultured once a week, with the media changed every 2 days. For the performed experiments, the cells were plated in 96-well plates, both for cell viability and sterol analysis purposes. For the assessment of endogenous sterol synthesis, we grew the cultures in defined medium without cholesterol and without lipids. This was achieved by using EMEM with the N2 supplement (Gibco cat # 17502048), 1×, and L-glutamine 2 mM. At the end of the incubation, we used Hoechst dye in all wells to count the total number of cells using the ImageXpress Pico (Molecular Devices, San Jose, CA, USA) and CellReporterXpress software package version 2.9.4. After removing the medium, the wells were rinsed twice with 1× PBS, and antioxidants (butylated hydroxytoluene (BHT) and triphenylphosphonium (TPP) were added to each well and then stored at −80 °C for lipid analysis. All samples were analyzed within 2 weeks of storage.

### 2.3. Primary Neuronal and Astrocytes Cultures

All animal experiments were approved by the UNMC IACUC Review Board. All the experiments were performed according to the established animal welfare standards. Dhcr7^T93M/T93M^ mice were a courtesy of FD Porter, NIH, Bethesda, MD [31]. These mice have a pathogenic variant in both *Dhcr7* alleles at c.278C>T corresponding to the human T93M missense mutation. Notably, this pathogenic variant of the *Dhcr7* gene is the most common missense pathogenic variant described in patients with SLOS [31,63]. Primary cortical neuronal cultures were obtained from E18 Dhcr7^T93M/T93M^ mice as previously described [29,64]. Briefly, the brain was placed in a pre-chilled HBSS solution (w/o Ca^2+^, w/o Mg^2+^), cerebral cortices were dissected, the meninges were removed, and the cortex was cut into small pieces. These small chunks were transferred to Trypsin/EDTA (0.25%) for 25 min at 37 °C. After this, the trypsin solution was replaced with the trypsin inhibitor solution, and the tissue was centrifuged for 5 min. This was followed by resuspending the tissue pieces in the Neurobasal^TM^ medium (Gibco cat # 21103049) with the B-27^TM^ supplement (Gibco cat # 17504044), 1×. The obtained small brain tissue chunks were triturated with a fire-polished Pasteur pipette. Cells were pelleted by centrifugation for 5 min at 80× *g*. The cell pellet was resuspended in the Neurobasal^TM^ medium with the B-27^TM^ supplement, and cell counts were obtained. These cells were plated on poly-D-lysine coated 96-well plate at density 70,000 cells/well. The growth medium consisted of the Neurobasal^TM^ medium with the B-27^TM^ supplement (Gibco #17504044) and GlutaMAX^TM^ (2 mM). Cells were kept at 37 °C with 5% CO_2_ for 6 days. Cells were exposed to HYZ every 48 h. At the end of the incubation, Hoechst dye was added to all wells in the 96-well plate. The total number of cells was calculated on ImageXpress Pico with the CellReporterXpress software package. After removing the medium, wells were rinsed twice with 1× PBS, and antioxidants (BHT plus TPP) were added to each well and then stored at −80 °C for lipid analysis. All samples were analyzed within 2 weeks of freezing.

We used the same source of cells for neuronal and glial cultures. For glial cultures, the cells were plated in 100 mm dishes at density of 10 × 10^6^ in Dulbecco’s modified Eagle’s medium (DMEM) with 10% fetal bovine serum (FBS, Seradigm cat # 97068-085). Under these conditions, astrocytes were adhered, divided, and fully populated 100 mm plate within 10–14 days. Once this was achieved, they were rinsed using the cold jet method [65]. Following this, the astrocytes were trypsinized and plated in 96-well plates in DMEM plus 10% FBS at 30,000 cells/well density. The next day, the medium was replaced, and astrocytes were grown in the Neurobasal^TM^ medium with the B-27^TM^ supplement without cholesterol (the medium was identical to the one used for neuronal cell cultures). Cells were incubated at 37 °C, 5% CO_2_ for 6 days, and were exposed to HYZ every 48 h. Following this incubation, cells were counted and processed for analysis with the same method as the neuronal cultures.

### 2.4. Human Dermal Fibroblast Cultures

Fibroblast cultures were also described in a previous publication [62]. All fibroblast cultures used for experiments were between passages 8 and 15. All cells were maintained in DMEM containing 25 mM glucose and 1 mM sodium pyruvate supplemented with 2 mM L-glutamine, 10% FBS (Seradigm cat # 97068-085), and 50 U/mL antibiotic (Penicillin-Streptomycin 5000 U/mL; Gibco cat # 15070063) at 37 °C and 5% CO_2_. For exposure to hydroxyzine (one dose every 48 h), cells were cultured in DMEM with 10% delipidated FBS in 96-well plates for 6 days. FBS (Seradigm cat # 97068-085) was delipidated according to the protocol described in Brovkovych et al. [66]. Briefly, 10 g of Fumed Silica (Sigma cat # S5130) was mixed with 500 mL of FBS overnight at +4 °C. The following day, mixture was centrifuged at 15,000× *g* for 30 min, and the supernatant (delipidated FBS) was stored in aliquots at −20 °C. After mixing delipidated FBS with the medium, the cell medium was filtered.

### 2.5. Post-Lanosterol LC-MS/MS Measurements

Sterol extractions were performed by adding a sterol standards mixture (10 µL) and MeOH (100 µL) to each of the 96-well plates [67]. A stock solution of deuterated standards was made containing 30 µM of *d_7_*-Chol, *d_6_*-Lan, *d_7_*-dHLan and 3 µM *d_7_*-7-DHC, *d_7_*-8-DHC, *d_6_*-Des, *d_6_*-7-DHD, *d_6_*-8-DHD, *d_7_*-Lath, *d_6_*-DHL, *d_7_*-Zyme, *d_6_*-Zym, *d_7_*-14d-Zyme, and *d_6_*-14d-Zym in MeOH. The standard stock solution contained 1% (*v*/*v*) Et_3_N and an antioxidant mixture (BHT 2.5 mg/mL and PPh_3_ 1 mg/mL in EtOH) to prevent isomerization and/or oxidation. To each well of the 96-well plate, the standard mixture (10 µL) and MeOH (100 µL) were added [67]. The plate was agitated for 30 min on a shaker and rested to settle the cell debris. The MeOH extract was dried under vacuum in an analysis plate. The freshly prepared derivatizing reagent consisted of 2-methyl-6-nitrobenzoic anhydride (20 mg), N,N-dimethylglycine (14 mg), DMAP (6 mg), and Et_3_N (0.1 mL) in anhydrous CHCl_3_ (0.9 mL). The derivatizing reagent (100 µL) was applied to each sample, and the reaction progressed at room temperature for 30 min. The vacuum-dried samples were subsequently dissolved in MeOH (100 µL) for LC-MS/MS analysis. Sample analysis was performed on an Acquity UPLC system equipped with ANSI-compliant well plate holder. Sterols (10 µL per injection) were analyzed on an Agilent Poroshell EC-C18 (10 cm × 2.1 mm, 1.9 µm) with CH_3_CN:MeOH:H_2_O, 70:25:5 (0.01% (v) formic acid, 1 mM NH_4_OAc) mobile phase at a column temperature of 40 °C. The flow rate was 400 µL/min with a total run time of 16 min. A TSQ Quantum tandem mass spectrometer (ThermoFisher) was used for MS detections, and data were acquired with a Xcalibur software package version 4.1. Selected reaction monitoring (SRM) of the DMG derivatives was acquired in the positive ion mode using electrospray ionization (ESI) [67].

### 2.6. Statistical Analyses

Statistical analyses were performed and visualized using Graphpad Prism 10 software package for Windows. Unpaired two-tailed *t*-tests were performed for individual comparisons between two groups. We utilized Welch’s correction when the variance between the two groups was significantly different. The *p* values for statistically significant differences are denoted in the legends: * 0.01 to 0.05; ** 0.001 to 0.01; *** 0.0001 to 0.001, and **** < 0.0001.

## 3. Results

### 3.1. HYZ Effects on Neuro2a Cells

**Figure 1:**
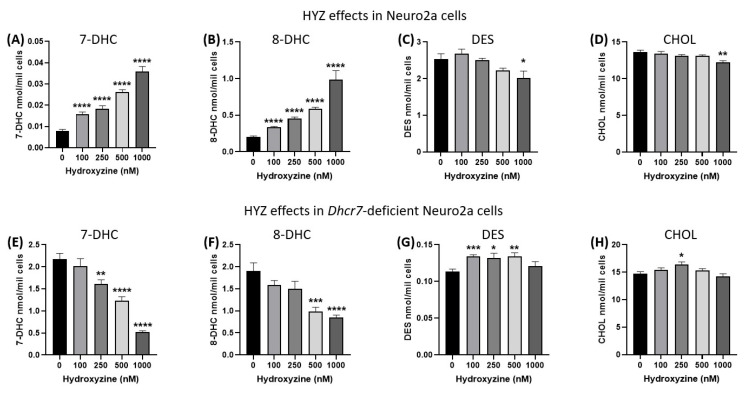
HYZ effects on wild-type and *Dhcr7*-defcient Neuro2a cells. Panels (**A**–**H**) represent LC-MS/MS measurements of six changed post-lanosterol intermediates. X-axes denote increasing concentrations of HYZ, and Y-axes denote sterol intermediate levels in nmol/million cells (n = 8–16 replicates for each HYZ concentration). Error bars represent SEM. Note the dose-dependent reduction of 7-DHC in the *Dhcr7*-deficient Neuro2a cells, with minimal changes in CHOL levels. Statistical significance * *p* < 0.05; ** *p* < 0.01; *** *p* < 0.001, **** *p* < 0.0001. Numerical values are presented in Table S1.

The control and *Dhcr7*-deficient Neuro2a cells [61] were exposed to a vehicle or serial concentrations of HYZ ranging from 100 to 1000 nM. In the sham-treated *Dhcr7*-deficient cultures, 7-DHC levels were higher by >200-fold over the genetically unmodified Neuro2a cells (Figure 1A vs. Figure 1E), mimicking the biochemical findings in SLOS patients [68,69]. Similarly, 8-DHC levels were approximately 10-fold higher in the sham-treated genetically modified *Dhcr7*-deficient Neuro2a cells than in the vehicle-treated controls (Figure 1B vs. Figure 1F). The baseline sham-treated sterol difference was also apparent in DES levels, as DES levels were approximately 8-fold lower in the *Dhcr7*-deficient cells (Figure 1C vs. Figure 1G).

In the control Neuro2a cells (Figure 1A–D, upper row of panels), LC-MS/MS analyses revealed a dose-dependent increase in 8-DHC of up to 300% at the highest tested HYZ dose (*p* < 0.0001 over baseline), coupled with a decrease of 20% in DES (*p* = 0.041 over baseline at the highest dose). CHOL levels were only slightly affected and only at the highest HYZ dose (10% decrease, *p* = 0.0017). In genetically unmodified Neuro2a cells, 7-DHC was increased by >400% (*p* < 0.0001).

In *Dhcr7*-deficient Neuro2a cells (Figure 1E–H, lower row panels), the post-lanosterol analyte profile was quite different in response to increasing concentrations of HYZ. 7-DHC levels in response to HYZ reported a dose-dependent decrease of up to 75% (2.2 to 0.52 nmol/mil cells) (*p* < 0.0001). Similarly, 8-DHC levels showed a dose-dependent decrease by up to 55% (1.9 to 0.8 nmol/mil cells) (*p* < 0.0001) in response to HYZ treatment. DES levels, in contrast, showed a slight increase of about 5–10% (*p* < 0.004 at c vs. 500 nM HYZ). CHOL levels remained virtually unchanged even when the *Dhcr7*-deficient Neuro2a cells were exposed to the highest concentration of HYZ.

In summary, the findings confirmed that *Dhcr7*-deficient Neuro2a cells recapitulated the biochemical SLOS findings in vitro and that HYZ was able to counteract the high levels of 7-DHC and 8-DHC without a further impediment of residual cholesterol levels.

### 3.2. HYZ Effects on Dhcr7^T93M/T93M^ Neurons and Glial Cells

**Figure 2:**
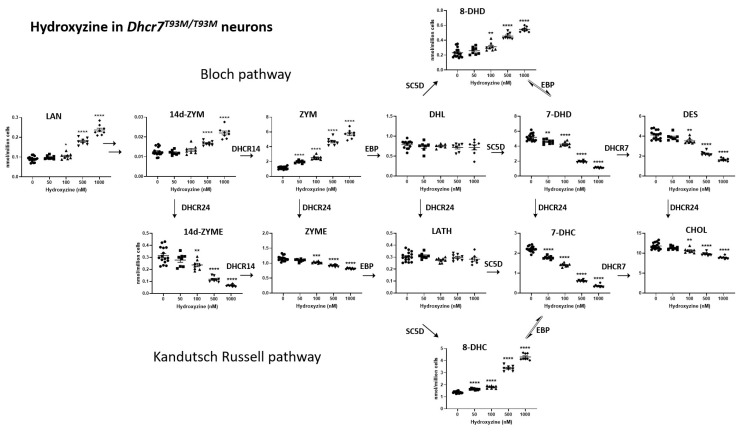
HYZ effects on Dhcr7^T93M/T93M^ neuronal cultures. Panels represent LC-MS/MS measurements of post-lanosterol intermediates. X-axes denote increasing concentrations of HYZ, and Y-axes denote sterol intermediate levels in nmol/million cells. Error bars represent SEM. Individual symbols correspond to replicates (n = 8–16 for each HYZ concentration). Arrows denote the biosynthetic pathway with corresponding enzymes. Note the strong decrease in 7-DHC and 7-DHD and the multiple post-lanosterol intermediates changed in a complex pattern. Statistical significance * *p* < 0.05; ** *p* < 0.01; *** *p* < 0.001, **** *p* < 0.0001. Numerical values are presented in Table S2.

Embryonic day 18 Dhcr7^T93M/T93M^ neurons [31,67], grown for 6 days in cultures, were exposed to vehicle or serial concentrations of HYZ ranging from 50 to 1000 nM. Glial cells, cultured 6 days, underwent a similar treatment. HYZ had a strong effect on post-lanosterol biosynthesis in developing neurons. The observed changes were dose-dependent and encompassed most of the tested analytes (Figure 2). In neurons, at the highest concentration, HYZ exposure resulted in increasing levels of LAN (262%, *p* < 0.0001), ZYM (496%, *p* < 0.0001), 8-DHC (311%, *p* < 0.0001), and 8-DHD (236%, *p* < 0.0001). In contrast, at the highest concentration, HYZ reduced levels of 7-DHC (84%, *p* < 0.0001), 7-DHD (78%, *p* < 0.0001), DES (60%, *p* < 0.0001), and ZYME (29%, *p* < 0.0001). CHOL levels only slightly decreased (25%, *p* < 0.0001), while LATH and DHL levels remained unchanged.

**Figure 3:**
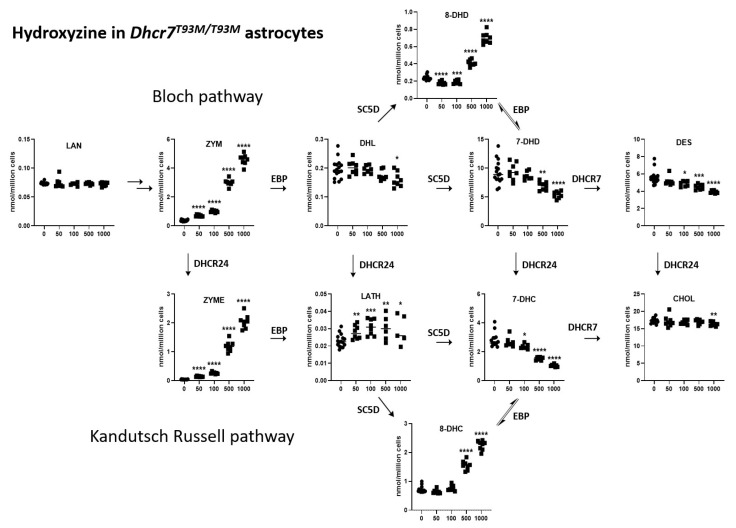
HYZ effects on Dhcr7^T93M/T93M^ astrocytes. Panels represent LC-MS/MS measurements of post-lanosterol intermediates. X-axes denote increasing concentrations of HYZ, and Y-axes denote sterol intermediate levels in nmol/million cells. Error bars represent SEM. Individual symbols correspond to replicates (n = 5–16 for each HYZ concentration). Arrows denote biosynthetic pathways with corresponding enzymes. Note the strong decrease in 7-DHC and 7-DHD and the multiple post-lanosterol intermediates changed in a complex pattern. Statistical significance * *p* < 0.05; ** *p* < 0.01; *** *p* < 0.001, **** *p* < 0.0001. Numerical values are presented in Table S3.

In glial cells (Figure 3), the highest concentration of HYZ exposure resulted in increasing levels of ZYM (1249%, *p* < 0.0001), ZYME (5000%, *p* < 0.0001), 8-DHC (315%, *p* < 0.0001), and 8-DHD (290%, *p* < 0.0001). In contrast, at the highest concentration, HYZ reduced levels of 7-DHC (63%, *p* < 0.0001), 7-DHD (42%, *p* < 0.0001), and DES (30%, *p* < 0.0001). CHOL levels only slightly decreased (6%, *p* < 0.0047). Thus, the most important difference between the HYZ response between neuronal and glial cultures was in the levels of ZYME, which reported a decrease in the neuronal cultures and a strong, dose-dependent increase in glial cultures. This might reflect differential sterol pathway utilization used in neurons and astrocytes.

In summary, HYZ showed a strong modulation of the post-lanosterol biosynthesis intermediates, which encompassed most of the tested analytes from both the Kandutsch–Russell (Figure 2) and Bloch (Figure 3) biosynthetic pathways. Notably, the neuronal and glial responses were somewhat different from those seen in the *Dhcr7*-deficient Neuro2a cells, suggesting intrinsic response differences between the in vitro SLOS models. The most notable difference is perhaps the response of 8-DHC levels, which decreased in the HYZ-treated *Dhcr7*-deficient Neuro2a cells and showed a dose-dependent increase in the Dhcr7^T93M/T93M^ neuronal and glial cultures. This finding might represent an intrinsic difference between the response of genetically modified neuroblastoma cell line vs. primary neurons and astrocytes. However, perhaps the most important, congruent finding between the three in vitro SLOS models was a strong, dose-dependent decrease in 7-DHC, the core biochemical hallmark of SLOS.

### 3.3. HYZ Effects on Human Dermal Fibroblasts (HDFs) from SLOS Patients and Controls

Three different HDF lines from SLOS patients and four HDF lines from controls [62,70] were grown for 6 days in cultures and were exposed to vehicle or concentrations of HYZ ranging from 100 to 500 nM.

**Figure 4:**
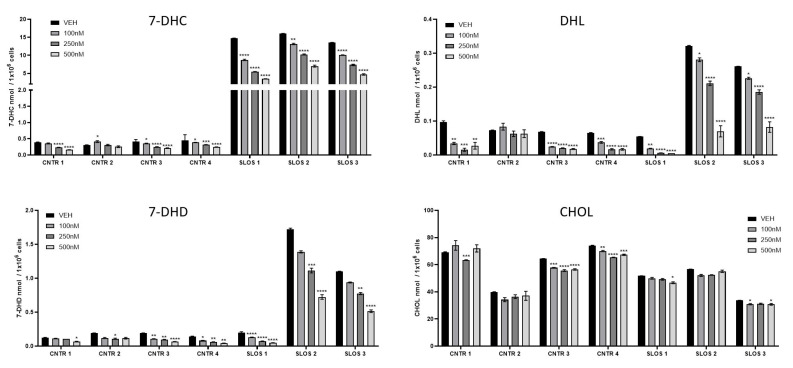
HYZ effects on human dermal fibroblasts from control and SLOS patients in the Bloch part of the post-lanosterol biosynthetic pathway. Panels represent LC-MS/MS measurements of post-lanosterol intermediates. X-axes denote increasing concentrations of HYZ, and Y-axes denote sterol intermediate levels in nmol/million cells (n = 3–6 technical replicates for each concentration of the 3 SLOS and 4 control cell lines). Arrows denote biosynthetic pathways with corresponding enzymes. Note the differences between control and SLOS fibroblast in analyte levels, and the strong, dose-dependent responses to HYZ. Statistical significance * *p* < 0.05; ** *p* < 0.01; *** *p* < 0.001, **** *p* < 0.0001. Numerical values are presented in Tables S4 and S5.

**Figure 5:**
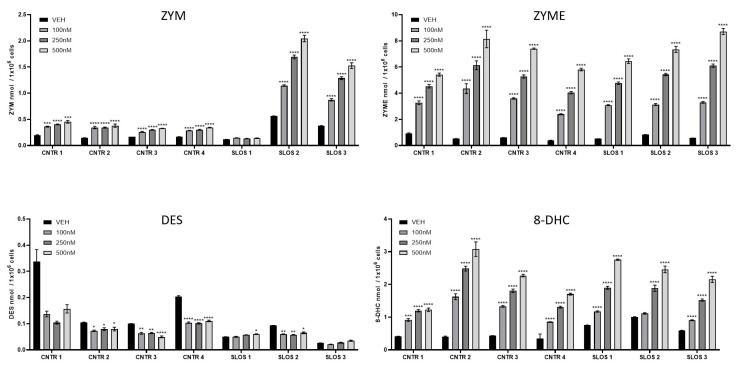
HYZ effects on human dermal fibroblasts from control and SLOS patients in the Kandutsch–Russell part of the post-lanosterol biosynthetic pathway. Panels represent LC-MS/MS measurements of post-lanosterol intermediates. X-axes denote increasing concentrations of HYZ, and Y-axes denote sterol intermediate levels in nmol/mil cells (n = 3–6 technical replicates for each concentration of the 3 SLOS and 4 control cell lines). Arrows denote biosynthetic pathways with corresponding enzymes. Note the differences between control and SLOS fibroblast in analyte levels and the strong dose-dependent responses to HYZ. Statistical significance * *p* < 0.05; ** *p* < 0.01; *** *p* < 0.001, **** *p* < 0.0001. Numerical values are presented in Tables S4 and S5.

Control HDFs had generally lower baseline levels of 7-DHC, 8-DHC, DHL, and 7-DHD in comparison to HDFs originating from SLOS patients (Figure 4 and Figure 5). Of these, the most marked differences without treatment were observed for 7-DHC, followed by 7-DHD and DHL. Baseline DES levels were decreased in HDFs from SLOS patients, while baseline CHOL levels showed variability across all HDFs (regardless of disease status) in the untreated cultures. HYZ altered sterol profile in the control HDFs in a dose-dependent manner. However, it is noteworthy that these sterol intermediate levels were very low.

HDFs originating from SLOS patients showed an even more robust post-lanosterol biochemical profile change in response to HYZ. 7-DHC, DHL, and 7-DHD were decreased, while ZYM, ZYME, and 8-DHC were increased. Notably, the treatment had no discernable effect on overall CHOL levels.

In summary, across the four in vitro SLOS models, the most consistent findings were strong and statistically significant decreases in 7-DHC and 7-DHD levels and increased ZYM levels. The other sterol intermediates, while robustly changed in one or two SLOS models as a result of HYZ treatment, reported a divergent directionality of change. This underscores the importance of obtaining convergent data and avoiding extrapolating conclusions based on a single in vitro model.

## 4. Discussion

Many prescription medications have a post-lanosterol biosynthesis modulatory effect [71,72,73,74]. These effects have primarily focused on the inhibition of DHCR7 in the context of 7-DHC and cholesterol level changes. Previous studies described DHCR7 inhibitory effects of aripiprazole, haloperidol, sertraline, cariprazine, and trazodone [67,75,76,77]. However, HYZ effects on sterol biosynthesis are much more complex and are clearly mediated through a different molecular mechanism.

Our findings demonstrate that HYZ is a strong modulator of post-lanosterol biosynthesis. In control Neuro2a cells, HYZ treatment resulted in altered levels of sterol post-lanosterol intermediates. This finding is already noteworthy, as the sterol biosynthesis modulating effect of HYZ has not been characterized to date. Knowing the fragile homeostatic balance of sterol intermediates and the essential role of cholesterol during development, this is a finding that should not be overlooked. Notably, the divergent biochemical response to HYZ between the genetically unmodified and Dhcr7^−/−^ cells in 7-DHC, 8-DHC, and DES levels is surprising and remains unexplained at the current time. We hypothesize that HYZ might act as a post-lanosterol “biosynthesis leveler”, reducing increased sterol precursors levels, and increasing low precursor levels. This might be mediated through a yet unknown biochemical feedback loop, either through a receptor-dependent mechanism or direct actions on biosynthesis enzymes. Furthermore, we do not know if this observation is specific to tissues and/or cell types. These and other questions should be addressed in follow-up experiments.

Previous studies reported that *DHCR7*^+/−^ individuals, making up approximately 3% of the human population, might be particularly susceptible to alterations in sterol biosynthesis when exposed the medications with sterol-inhibiting side effects [78,79,80]. Based on this, HYZ should be potentially avoided in *DHCR7*^+/−^ healthy mothers during pregnancy, as they might carry a child with a similar genotype, potentially leading to cumulative risk for adverse effects.

However, in contrast to all our previous studies of medications altering sterol biosynthesis, HYZ has unusual effects. Namely, the observed differences in the HYZ-altered post-lanosterol biochemical profile across the *Dhcr7*-deficient Neuro2a cells, Dhcr7^T93M/T93M^ mouse neurons and glial cells, and human dermal fibroblasts from SLOS patients are surprising, as all four of these in vitro systems use the same sterol biosynthesis machinery. In particular, the 8-DHC decrease in HYZ-treated *Dhcr7*-deficient Neuro2a cells and an increase in 8-DHC in the other three SLOS models suggests a different yet unknown modulatory system. One possible explanation for this is that HYZ does not act directly on the sterol biosynthesis enzymes but through a gene expression mechanism, which is very tissue-specific.

**Figure 6:**
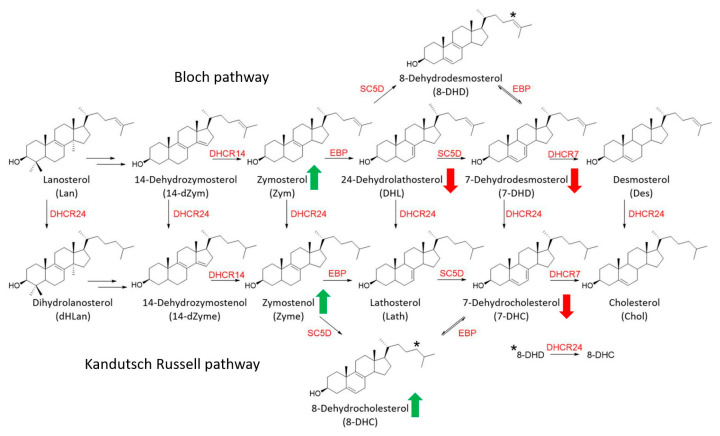
Summary of HYZ action on post-lanosterol biosynthesis in human dermal fibroblasts from SLOS patients. Only chemical structures of sterols analyzed by LC-MS/MS are shown. * identifies conversion of 8-DHD double bond to 8-DHC, catalyzed by the DHCR24 enzyme. Enzymes are denoted in red, analytes in black, and arrows correspond to statistically significant increases (green) and decreases (red). Note that the observed sterol intermediate increases and decreases can be potentially explained by the inhibition of EBP, which leads to the accumulation of substrates (ZYM, ZYME, and 8-DHC) and reduction in products (DHL, 7-DHD, and 7-DHC).

At the molecular level, the explanation for the complex alteration profile of post-lanosterol biosynthesis is a potential (direct or indirect) EBP inhibition seen in Dhcr7^T93M/T93M^ mouse neurons, glial cells, and human dermal fibroblasts from SLOS patients. With EBP inhibition, its substrates ZYM and ZYME would accumulate (see Figure 6), and the higher ZYME levels would also result in elevated 8-DHC levels. Thus, the downstream levels of DHL, 7-DHD, and 7-DHC would be reduced. However, this EBP inhibition (or potentially reduced EBP expression levels) by HYZ would have to be tested in future follow-up experiments, beyond the scope of the current studies.

Another important question, raised by our studies, refers to the potential benefit of HYZ to SLOS patients. Human epidemiological studies clearly indicate that the DHCR7 inhibitors can be considered teratogens [47]. In this context, reductions in DHL, 7-DHD, and 7-DHC levels would suggest that HYZ should be considered as a potential therapeutic agent for patients with SLOS, as it would reduce the generation of toxic oxysterols without further compromising cholesterol levels. However, this argument is significantly weakened by the observed rise in ZYM, ZYME, and 8-DHC levels in the fibroblast of SLOS patients. Currently, we have a limited understanding of the oxysterols they might generate and their effects on human development and overall health. Notably, pathogenic variants in the EBP gene have been found to cause X-linked chondrodysplasia punctata 2 [81,82], a developmental disability sharing some phenotypic features with SLOS. In this context, if the post-lanosterol profile we observed is a result of EBP inhibition, the therapeutic use of HYZ in SLOS patients might not be ideal.

Still, the ultimate utility of HYZ as a therapeutic compound for SLOS patients will depend on a complex set of factors: dose, timing of treatment, beneficial reduction in 7-DHC levels, magnitude of potentially detrimental rise in 8-DHC levels, clearance speed of sterol intermediates/oxysterols, and other variables. One could envision that exposure to HYZ during embryonic development could be detrimental, yet potentially beneficial during later stages of life. Finally, based on all these variables and the divergence of sterol data across the models we observed, can HYZ be beneficial for one organ system in SLOS, but detrimental for another? These critical questions will have to be addressed in follow-up experiments.

## Data Availability

Data generated in this series of experiments will be deposited to the NCBI database at the time of acceptance to publication.

## Supplementary Materials

The following supporting information can be downloaded at: https://www.mdpi.com/article/10.3390/biom15040562/s1. Table S1: LC-MS/MS measurements of sterol intermediates in wild-type and *Dhcr7*-deficient Neuro2a cells; Table S2: LC-MS/MS measurements of sterol intermediates in cultured Dhcr7^T93M/T93M^ neurons; Table S3: LC-MS/MS measurements of sterol intermediates in cultured Dhcr7^T93M/T93M^ astrocytes; Table S4: LC-MS/MS measurements of sterol intermediates in cultured human dermal fibroblasts from control subjects; Table S5: LC-MS/MS measurements of sterol intermediates in cultured human dermal fibroblasts from SLOS patients.

## Abbreviations

SLOS	Smith–Lemli–Opitz syndrome
HYZ	hydroxyzine
DHCR7	Dehydrocholesterol reductase 7
7-DHC	7-dehydrocholesterol
8-DHC	8-dehydrocholesterol
DES	desmosterol
LATH	lathosterol
LAN	lanosterol
ZYM	zymosterol
ZYME	zymostenol
DHL	dehydrolathosterol
7-DHD	7-dehydrodesmosterol
8-DHD	8-dehydrodesmosterol
SC5D	sterol-C5-desaturase
EBP	emopamil-binding protein
DHCR24	Dehydrocholesterol reductase 24

## References

[1] Porter F.D. (2000). RSH/Smith-Lemli-Opitz syndrome: A multiple congenital anomaly/mental retardation syndrome due to an inborn error of cholesterol biosynthesis. Mol. Genet. Metab..

[2] Smith D.W., Lemli L., Opitz J.M. (1964). A Newly Recognized Syndrome of Multiple Congenital Anomalies. J. Pediatr..

[3] Sanghera A.S., Zeppieri M. (2025). Smith-Lemli-Opitz Syndrome.

[4] Yilmaz M., Bebek O., Turkyilmaz A. (2024). Smith-Lemli-Opitz Syndrome with Biallelic c.1295A>G (p.Tyr432Cys) Variant in the DHCR7 Gene in a 73-Year-Old Woman: Report of the Oldest Patient. Mol. Syndromol..

[5] Westbye A.B., Dizdarevic L.L., Dahl S.R., Asprusten E.A., Bliksrud Y.T., Sandblom A.L., Diczfalusy U., Thorsby P.M., Retterstøl K. (2024). A sterol panel for rare lipid disorders: Sitosterolemia, cerebrotendinous xanthomatosis, and Smith-Lemli-Opitz syndrome. J. Lipid Res..

[6] Bianconi S.E., Cross J.L., Wassif C.A., Porter F.D. (2015). Pathogenesis, Epidemiology, Diagnosis and Clinical Aspects of Smith-Lemli-Opitz Syndrome. Expert. Opin. Orphan Drugs.

[7] Coupe S., Hertzog A., Foran C., Tolun A.A., Suthern M., Chung C.W.T., Ellaway C. (2023). Keeping you on your toes: Smith-Lemli-Opitz Syndrome is an easily missed cause of developmental delays. Clin. Case Rep..

[8] Sanchez-Soler M.J., Serrano-Anton A.T., Lopez-Gonzalez V., Ballesta-Martinez M.J., Guillen-Navarro E. (2022). Extremely variable expressivity in Smith-Lemli-Opitz syndrome: Review of 4 cases. Pediatr Engl. Ed..

[9] Rozdzynska-Swiatkowska A., Ciara E., Halat-Wolska P., Krajewska-Walasek M., Jezela-Stanek A. (2021). Anthropometric characteristics of 65 Polish Smith-Lemli-Opitz patients. J. Appl. Genet..

[10] Park J.E., Lee T., Ha K., Ki C.S. (2021). Carrier frequency and incidence estimation of Smith-Lemli-Opitz syndrome in East Asian populations by Genome Aggregation Database (gnomAD) based analysis. Orphanet J. Rare Dis..

[11] Lund E., Starck L., Venizelos N. (1996). Detection of defective 3 beta-hydroxysterol delta 7-reductase activity in cultured human fibroblasts: A method for the diagnosis of Smith-Lemli-Opitz syndrome. J. Inherit. Metab. Dis..

[12] Haas D., Armbrust S., Haas J.P., Zschocke J., Muhlmann K., Fusch C., Neumann L.M. (2005). Smith-Lemli-Opitz syndrome with a classical phenotype, oesophageal achalasia and borderline plasma sterol concentrations. J. Inherit. Metab. Dis..

[13] Dallaire L., Mitchell G., Giguere R., Lefebvre F., Melancon S.B., Lambert M. (1995). Prenatal diagnosis of Smith-Lemli-Opitz syndrome is possible by measurement of 7-dehydrocholesterol in amniotic fluid. Prenat. Diagn..

[14] Tint G.S., Seller M., Hughes-Benzie R., Batta A.K., Shefer S., Genest D., Irons M., Elias E., Salen G. (1995). Markedly increased tissue concentrations of 7-dehydrocholesterol combined with low levels of cholesterol are characteristic of the Smith-Lemli-Opitz syndrome. J. Lipid Res..

[15] Shefer S., Salen G., Batta A.K., Honda A., Tint G.S., Irons M., Elias E.R., Chen T.C., Holick M.F. (1995). Markedly inhibited 7-dehydrocholesterol-delta 7-reductase activity in liver microsomes from Smith-Lemli-Opitz homozygotes. J. Clin. Investig..

[16] Kelley R.I. (1995). Diagnosis of Smith-Lemli-Opitz syndrome by gas chromatography/mass spectrometry of 7-dehydrocholesterol in plasma, amniotic fluid and cultured skin fibroblasts. Clin. Chim. Acta.

[17] Honda A., Tint G.S., Salen G., Batta A.K., Chen T.S., Shefer S. (1995). Defective conversion of 7-dehydrocholesterol to cholesterol in cultured skin fibroblasts from Smith-Lemli-Opitz syndrome homozygotes. J. Lipid Res..

[18] Xu L., Korade Z., Rosado D.A., Liu W., Lamberson C.R., Porter N.A. (2011). An oxysterol biomarker for 7-dehydrocholesterol oxidation in cell/mouse models for Smith-Lemli-Opitz syndrome. J. Lipid Res..

[19] Xu L., Liu W., Sheflin L.G., Fliesler S.J., Porter N.A. (2011). Novel oxysterols observed in tissues and fluids of AY9944-treated rats: A model for Smith-Lemli-Opitz syndrome. J. Lipid Res..

[20] Xu L., Sheflin L.G., Porter N.A., Fliesler S.J. (2012). 7-Dehydrocholesterol-derived oxysterols and retinal degeneration in a rat model of Smith-Lemli-Opitz syndrome. Biochim. Biophys. Acta.

[21] Yin H., Xu L., Porter N.A. (2011). Free radical lipid peroxidation: Mechanisms and analysis. Chem. Rev..

[22] Liu W., Xu L., Lamberson C.R., Merkens L.S., Steiner R.D., Elias E.R., Haas D., Porter N.A. (2013). Assays of plasma dehydrocholesteryl esters and oxysterols from Smith-Lemli-Opitz syndrome patients. J. Lipid Res..

[23] Shinkyo R., Xu L., Tallman K.A., Cheng Q., Porter N.A., Guengerich F.P. (2011). Conversion of 7-dehydrocholesterol to 7-ketocholesterol is catalyzed by human cytochrome P450 7A1 and occurs by direct oxidation without an epoxide intermediate. J. Biol. Chem..

[24] Xu L., Korade Z., Rosado D.A., Mirnics K., Porter N.A. (2013). Metabolism of oxysterols derived from nonenzymatic oxidation of 7-dehydrocholesterol in cells. J. Lipid Res..

[25] Francis K.R., Ton A.N., Xin Y., O’Halloran P.E., Wassif C.A., Malik N., Williams I.M., Cluzeau C.V., Trivedi N.S., Pavan W.J. (2016). Modeling Smith-Lemli-Opitz syndrome with induced pluripotent stem cells reveals a causal role for Wnt/beta-catenin defects in neuronal cholesterol synthesis phenotypes. Nat. Med..

[26] Freel B.A., Kelvington B.A., Sengupta S., Mukherjee M., Francis K.R. (2022). Sterol dysregulation in Smith-Lemli-Opitz syndrome causes astrocyte immune reactivity through microglia crosstalk. Dis. Model. Mech..

[27] Korade Z., Xu L., Shelton R., Porter N.A. (2010). Biological activities of 7-dehydrocholesterol-derived oxysterols: Implications for Smith-Lemli-Opitz syndrome. J. Lipid Res..

[28] Pfeffer B.A., Xu L., Fliesler S.J. (2021). Transcriptomic Changes Associated with Loss of Cell Viability Induced by Oxysterol Treatment of a Retinal Photoreceptor-Derived Cell Line: An In Vitro Model of Smith-Lemli-Opitz Syndrome. Int. J. Mol. Sci..

[29] Xu L., Mirnics K., Bowman A.B., Liu W., Da J., Porter N.A., Korade Z. (2012). DHCEO accumulation is a critical mediator of pathophysiology in a Smith-Lemli-Opitz syndrome model. Neurobiol. Dis..

[30] Gaoua W., Chevy F., Roux C., Wolf C. (1999). Oxidized derivatives of 7-dehydrocholesterol induce growth retardation in cultured rat embryos: A model for antenatal growth retardation in the Smith-Lemli-Opitz syndrome. J. Lipid Res..

[31] Correa-Cerro L.S., Wassif C.A., Kratz L., Miller G.F., Munasinghe J.P., Grinberg A., Fliesler S.J., Porter F.D. (2006). Development and characterization of a hypomorphic Smith-Lemli-Opitz syndrome mouse model and efficacy of simvastatin therapy. Hum. Mol. Genet..

[32] Li A., Hines K.M., Ross D.H., MacDonald J.W., Xu L. (2022). Temporal changes in the brain lipidome during neurodevelopment of Smith-Lemli-Opitz syndrome mice. Analyst.

[33] Thurm A., Tierney E., Farmer C., Albert P., Joseph L., Swedo S., Bianconi S., Bukelis I., Wheeler C., Sarphare G. (2016). Development, behavior, and biomarker characterization of Smith-Lemli-Opitz syndrome: An update. J. Neurodev. Disord..

[34] Tomita H., Hines K.M., Herron J.M., Li A., Baggett D.W., Xu L. (2022). 7-Dehydrocholesterol-derived oxysterols cause neurogenic defects in Smith-Lemli-Opitz syndrome. eLife.

[35] Li A., Tomita H., Xu L. (2023). Temporal gene expression changes and affected pathways in neurodevelopment of a mouse model of Smith-Lemli-Opitz syndrome. bioRxiv.

[36] Li A., Xu L. (2023). MALDI-IM-MS Imaging of Brain Sterols and Lipids in a Mouse Model of Smith-Lemli-Opitz Syndrome. bioRxiv.

[37] Gabor K., Mesev E.V., Madenspacher J., Meacham J., Rai P., Moon S., Wassif C.A., Shaikh S.R., Tucker C., Karmaus P. (2024). Sterol biosynthesis regulates TLR signaling and the innate immune response in a Smith-Lemli-Opitz syndrome model. J. Clin. Investig..

[38] Dietschy J.M. (2009). Central nervous system: Cholesterol turnover, brain development and neurodegeneration. Biol. Chem..

[39] Dietschy J.M., Turley S.D. (2001). Cholesterol metabolism in the brain. Curr. Opin. Lipidol..

[40] Bjorkhem I., Meaney S. (2004). Brain cholesterol: Long secret life behind a barrier. Arterioscler. Thromb. Vasc. Biol..

[41] Mitsche M.A., McDonald J.G., Hobbs H.H., Cohen J.C. (2015). Flux analysis of cholesterol biosynthesis in vivo reveals multiple tissue and cell-type specific pathways. eLife.

[42] Genaro-Mattos T.C., Anderson A., Allen L.B., Korade Z., Mirnics K. (2019). Cholesterol Biosynthesis and Uptake in Developing Neurons. ACS Chem. Neurosci..

[43] Tierney E., Conley S.K., Goodwin H., Porter F.D. (2010). Analysis of short-term behavioral effects of dietary cholesterol supplementation in Smith-Lemli-Opitz syndrome. Am. J. Med. Genet. A.

[44] Wassif C.A., Kratz L., Sparks S.E., Wheeler C., Bianconi S., Gropman A., Calis K.A., Kelley R.I., Tierney E., Porter F.D. (2017). A placebo-controlled trial of simvastatin therapy in Smith-Lemli-Opitz syndrome. Genet. Med..

[45] Elias E.R., Orth L.E., Li A., Xu L., Jones S.M., Rizzo W.B. (2024). Cholic acid increases plasma cholesterol in Smith-Lemli-Opitz syndrome: A pilot study. Mol. Genet. Metab. Rep..

[46] Xu G., Salen G., Shefer S., Ness G.C., Chen T.S., Zhao Z., Salen L., Tint G. (1995). Treatment of the cholesterol biosynthetic defect in Smith-Lemli-Opitz syndrome reproduced in rats by BM 15.766. Gastroenterology.

[47] Starck L., Lovgren-Sandblom A., Bjorkhem I. (2002). Cholesterol treatment forever? The first Scandinavian trial of cholesterol supplementation in the cholesterol-synthesis defect Smith-Lemli-Opitz syndrome. J. Intern. Med..

[48] Korade Z., Kim H.Y., Tallman K.A., Liu W., Koczok K., Balogh I., Xu L., Mirnics K., Porter N.A. (2016). The Effect of Small Molecules on Sterol Homeostasis: Measuring 7-Dehydrocholesterol in Dhcr7-Deficient Neuro2a Cells and Human Fibroblasts. J. Med. Chem..

[49] Burgazli C.R., Rana K.B., Brown J.N., Tillman F. (2023). Efficacy and safety of hydroxyzine for sleep in adults: Systematic review. Hum. Psychopharmacol..

[50] Ferreri M., Hantouche E.G. (1998). Recent clinical trials of hydroxyzine in generalized anxiety disorder. Acta Psychiatr. Scand. Suppl..

[51] Guaiana G., Barbui C., Cipriani A. (2010). Hydroxyzine for generalised anxiety disorder. Cochrane Database Syst. Rev..

[52] Kim T., Kim K., Kim S., Kim J. (2022). Safety of hydroxyzine in the sedation of pediatric dental patients. J. Dent. Anesth. Pain. Med..

[53] ClinCalc (2022). Hydroxyzine Drug Usage Statistics, United States, 2013–2022.

[54] Gengo F.M., Dabronzo J., Yurchak A., Love S., Miller J.K. (1987). The relative antihistaminic and psychomotor effects of hydroxyzine and cetirizine. Clin. Pharmacol. Ther..

[55] Jotaki S., Murotani K., Hiraki T. (2024). Preventive Effect of Hydroxyzine on Postoperative Nausea and Vomiting: A Single-Center, Retrospective, Observational Cohort Study. J. Clin. Med..

[56] Akhondzadeh S. (2003). Hydroxyzine may be safe and effective in generalised anxiety disorder. Evid. Based Ment. Health.

[57] Llorca P.M., Spadone C., Sol O., Danniau A., Bougerol T., Corruble E., Faruch M., Macher J.-P., Sermet E., Servant D. (2002). Efficacy and safety of hydroxyzine in the treatment of generalized anxiety disorder: A 3-month double-blind study. J. Clin. Psychiatry.

[58] Paton D.M., Webster D.R. (1985). Clinical pharmacokinetics of H1-receptor antagonists (the antihistamines). Clin. Pharmacokinet..

[59] Simons F.E., Simons K.J., Frith E.M. (1984). The pharmacokinetics and antihistaminic of the H1 receptor antagonist hydroxyzine. J. Allergy Clin. Immunol..

[60] Foye W.O., Lemke T.L., Williams D.A. (2013). Foye’s Principles of Medicinal Chemistry.

[61] Korade Z., Kenworthy A.K., Mirnics K. (2009). Molecular consequences of altered neuronal cholesterol biosynthesis. J. Neurosci. Res..

[62] Korade Z., Xu L., Harrison F.E., Ahsen R., Hart S.E., Folkes O.M., Mirnics K., Porter N.A. (2014). Antioxidant supplementation ameliorates molecular deficits in Smith-Lemli-Opitz syndrome. Biol. Psychiatry.

[63] Correa-Cerro L.S., Porter F.D. (2005). 3beta-hydroxysterol Delta7-reductase and the Smith-Lemli-Opitz syndrome. Mol. Genet. Metab..

[64] Korade Z., Mi Z., Portugal C., Schor N.F. (2007). Expression and p75 neurotrophin receptor dependence of cholesterol synthetic enzymes in adult mouse brain. Neurobiol. Aging.

[65] Goudriaan A., Camargo N., Carney K.E., Oliet S.H., Smit A.B., Verheijen M.H. (2014). Novel cell separation method for molecular analysis of neuron-astrocyte co-cultures. Front. Cell Neurosci..

[66] Brovkovych V., Aldrich A., Li N., Atilla-Gokcumen G.E., Frasor J. (2019). Removal of Serum Lipids and Lipid-Derived Metabolites to Investigate Breast Cancer Cell Biology. Proteomics.

[67] Tallman K.A., Allen L.B., Klingelsmith K.B., Anderson A., Genaro-Mattos T.C., Mirnics K., Porter N.A., Korade Z. (2021). Prescription Medications Alter Neuronal and Glial Cholesterol Synthesis. ACS Chem. Neurosci..

[68] Bukelis I., Porter F.D., Zimmerman A.W., Tierney E. (2007). Smith-Lemli-Opitz syndrome and autism spectrum disorder. Am. J. Psychiatry.

[69] Haas D., Garbade S.F., Vohwinkel C., Muschol N., Trefz F.K., Penzien J.M., Zschocke J., Hoffmann G.F., Burgard P. (2007). Effects of cholesterol and simvastatin treatment in patients with Smith-Lemli-Opitz syndrome (SLOS). J. Inherit. Metab. Dis..

[70] Korade Z., Genaro-Mattos T.C., Tallman K.A., Liu W., Garbett K.A., Koczok K., Balogh I., Mirnics K., Porter N.A. (2017). Vulnerability of DHCR7(+/−) mutation carriers to aripiprazole and trazodone exposure. J. Lipid Res..

[71] Balog M., Anderson A.C., Heffer M., Korade Z., Mirnics K. (2022). Effects of Psychotropic Medication on Somatic Sterol Biosynthesis of Adult Mice. Biomolecules.

[72] Kim H.Y., Korade Z., Tallman K.A., Liu W., Weaver C.D., Mirnics K., Porter N.A. (2016). Inhibitors of 7-Dehydrocholesterol Reductase: Screening of a Collection of Pharmacologically Active Compounds in Neuro2a Cells. Chem. Res. Toxicol..

[73] Korade Z., Heffer M., Mirnics K. (2022). Medication effects on developmental sterol biosynthesis. Mol. Psychiatry.

[74] Peeples E.S., Mirnics K., Korade Z. (2024). Chemical Inhibition of Sterol Biosynthesis. Biomolecules.

[75] Genaro-Mattos T.C., Allen L.B., Anderson A., Tallman K.A., Porter N.A., Korade Z., Mirnics K. (2019). Maternal aripiprazole exposure interacts with 7-dehydrocholesterol reductase mutations and alters embryonic neurodevelopment. Mol. Psychiatry.

[76] Korade Z., Allen L.B., Anderson A., Tallman K.A., Genaro-Mattos T.C., Porter N.A., Mirnics K. (2021). Trazodone effects on developing brain. Transl. Psychiatry.

[77] Korade Z., Liu W., Warren E.B., Armstrong K., Porter N.A., Konradi C. (2017). Effect of psychotropic drug treatment on sterol metabolism. Schizophr. Res..

[78] Boland M.R., Tatonetti N.P. (2016). Investigation of 7-dehydrocholesterol reductase pathway to elucidate off-target prenatal effects of pharmaceuticals: A systematic review. Pharmacogenom. J..

[79] Cross J.L., Iben J., Simpson C.L., Thurm A., Swedo S., Tierney E., Bailey-Wilson J., Biesecker L., Porter F., Wassif C. (2015). Determination of the allelic frequency in Smith-Lemli-Opitz syndrome by analysis of massively parallel sequencing data sets. Clin. Genet..

[80] Lalovic A., Merkens L., Russell L., Arsenault-Lapierre G., Nowaczyk M.J., Porter F.D., Steiner R.D., Turecki G. (2004). Cholesterol metabolism and suicidality in Smith-Lemli-Opitz syndrome carriers. Am. J. Psychiatry.

[81] Ikegawa S., Ohashi H., Ogata T., Honda A., Tsukahara M., Kubo T., Kimizuka M., Shimode M., Hasegawa T., Nishimura G. (2000). Novel and recurrent EBP mutations in X-linked dominant chondrodysplasia punctata. Am. J. Med. Genet..

[82] Kumble S., Savarirayan R., Adam M.P., Feldman J., Mirzaa G.M., Pagon R.A., Wallace S.E., Amemiya A. (1993). Chondrodysplasia Punctata 2, X.-Linked. GeneReviews®.

